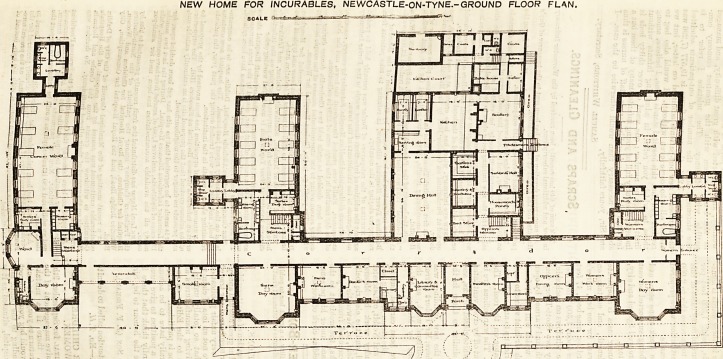# New Home for Incurables, Newcastle-On-Tyne

**Published:** 1893-10-21

**Authors:** 


NEW HOME FOR INCURABLES, NEWCASTLE-ON-TYNE.—GROUND FLOOR PLAN.

### HOSPITAL CONSTRUCTION.

### NEW HOME FOR INCURABLES, NEWCASTLE-ON-TYNE.

The plan of this institution, which we illustrate to-day, is of the pavilion type. The total accommodation is for sixty-eight patients, of whom twenty are cancer patients, and are housed in a separate wing. The main front faces south, and the various sitting-rooms for the staff and day-rooms for the patients are properly disposed on this side. The plan is simple, and generally well thought out. On the ground floor a spacious corridor traverses the building from east to west, having on its south side the day-rooms for patients and sitting-rooms and offices for the staff. The large wing at the back contains the general dining-hall for patients of both sexes, and the kitchen offices and stores. This wing is, except for the small part taken up by the officers' staircase, one storey only in height. The various offices are well arranged and compact, without being in any way restricted as to space. In the front building is a lift for conveying infirm patients from the ground floor to the first floor. The position of this lift is one of the few blots in an otherwise excellent plan. It is enclosed by walls on three sides, and is only open to the corridor on one of its narrower sides. It is curious how seldom architects recognize how objectionable it is to shut in a lift and its gearing in this way. The proper place for a lift is where it can be seen from at least three sides, and where no enclosure, except what may be needful for protection against accident, is required. The ward pavilions on either side of the administration block are two stories in height, and each contain two wards of ten beds each. The sanitary offices are placed in projecting towers communicating by cross-ventilated lobbies, both with the wards and with the dressing-room for the patients, a well contrived and convenient arrangement which we do not remember to have seen elsewhere. The wards give to each patient about 1,100 cubic feet, a fairly liberal allowance in a home of this nature, where but few of the patients are confined to bed by day. The cancer pavilion is a separate building of two storeys, and contains on each floor a large ward for nine beds and a separation ward for one bed. The latter is a most desirable arrangement in view of the peculiar disease treated here. The cubic space in these wards is somewhat greater than in the other wards, being 1,500 feet per head. Considering the offensive nature of many cases of this disease we should have been inclined to increase this to 2,000 feet. The designs for this excellent institution were prepared by Mr. Edward Shewbrooks, of Newcastle.

All the staircases throughout have York stone steps and landings, the outer end of the steps being supported on cast iron strings where necessary. They are well lighted and airy, and warmed by means of steam radiators.

The pavilion for the care of 20 cancer cases is a separate block two storeys high, 92 feet in length, and contains on each floor a day-room, a separation ward for one bed, nurse's duty-room, dressing-room, and a ward for nine beds, 47 feet long and 24 feet wide, which provides about 1,500 cubic feet of air space per bed. The wards have windows on each side, and they will be lighted artificially by two Stott-Thorp ventilating gas lights of four burners each, and warmed by open fires and ventilating steam radiators, which will admit fresh warmed air as desired; there is a fresh air inlet valve to each bed, and foul air extraction shafts carried up to and connected with Kite's automatic exhaust ventilator on the roof. The walls are finished with Parian cement. The ward offices are in an annex at the end of the pavilion, cut off by cross ventilated lobbies. The walls are lined with white glazed bricks, and the fittings and appurtenances are similar to those in the other annexes already described.

The contract for the erection of the new buildings was entrusted to Messrs. Middlemiss Brothers; Mr. Robert Herron has executed the plumbers and gas-fitters' work, and the painting and glazing have been done by Messrs. Adam Robertson and Son.

The ventilating arrangements have been carried out by Messrs. C. Kite and Co., of London, the steam heating and hot water supply by Messrs. Ashwell and Nesbit, of Leicester and London, the cooking and laundry work by Messrs. Thomas Bradford and Co., of Salford, and the electric bells and lightning conductors by Mr. F. Reid, of Newcastle.

The whole has been designed and executed from the plans prepared by Mr. Edward Shewbrooks, the architect to the Schools and Charities Committee, and under his personal supervision, assisted by Mr. David Reid as clerk of works.

## Figures and Tables

**Figure f1:**